# Equipment repaired is equipment gained

**Published:** 2009-06

**Authors:** V Srinivasan

**Affiliations:** Aravind Eye Care System Madurai, India v.srinivasan@aravind.org

According to WHO, at any one time, around 50 per cent of medical equipment in low-and middle-income countries cannot be used because of lack of maintenance or spare parts.^1^ We have found that unwillingness or inability to repair equipment and put it back in use is also a major cause.

New equipment is made available by different international non-government organisations (INGOs) competing with one another to help users. However, these organisations often do not support users with repairs, nor do they train users in maintenance and repair.

What happens to pieces of equipment that do not work? Usually they remain in the hospital, occupying space needed for patient care; they are sometimes abandoned in open spaces and exposed to the elements.

In 2007, the author was part of a team that ran a two-week biomedical workshop organised by an INGO in a low-income country. On the first day, the author noticed an operating table lying near the hospital garbage pit (Figure 1). He thought something should be done about it before the end of the workshop. On a closer examination the table was found to be of Japanese make, donated to the hospital under a bilateral cooperation programme. It was covered in dust and weeds had grown over it. The base and body were made of thick stainless steel so that the equipment had not rusted, though it had been there for more than a year.

On the eighth working day a suggestion was made to the trainees that they should try putting the table back to use. Eight volunteered but one thought it a waste of time to work on the table, which had clearly been abandoned for a long time.

The table was pulled out of the mud and weeds and shifted to a hard surface. That was a difficult job but heavy rain on the previous night had softened the ground which made the task easier.

**Figure:**
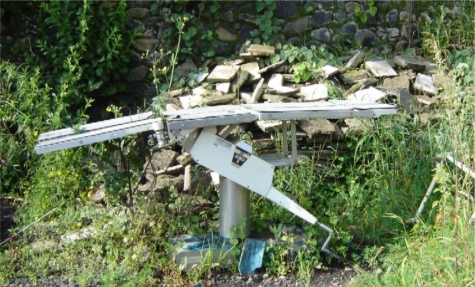
Figure 1. An operating table lies abandoned outside hospital in a low-income country.

V Srinivasan

We then tried to find out why the table had been abandoned. After a thorough wash with water to remove all the dust and dirt that had accumulated on it, the table was examined. The table could not be raised or lowered as designed, and the obvious conclusion was that the hydraulic system was not functioning. We opened the cylinder that should have oil it, but found it to be empty.

It is likely that the oil had drained drop by drop and no one had taken serious note of it. Eventually, when the oil level was low, the table had no longer moved up and down. The ‘broken’ table had then been removed from the operating theatre and left in the open, where the remaining oil had drained completely.

Next, the author and trainees dismantled the operating table part by part and shifted it to the workshop. In the workshop, the parts of the table were cleaned further and all moving parts were oiled. The gasket (the rubber ring which seals the system) had dried out because the table had been left outside for many months; we oiled it to ensure that the hydraulic system would once again have an airtight seal. We filled the hydraulic system with lubricating oil (poured into the cylinder using a funnel). When assembled, all other functions of the table were found to work as designed (Figure 2).

**Figure:**
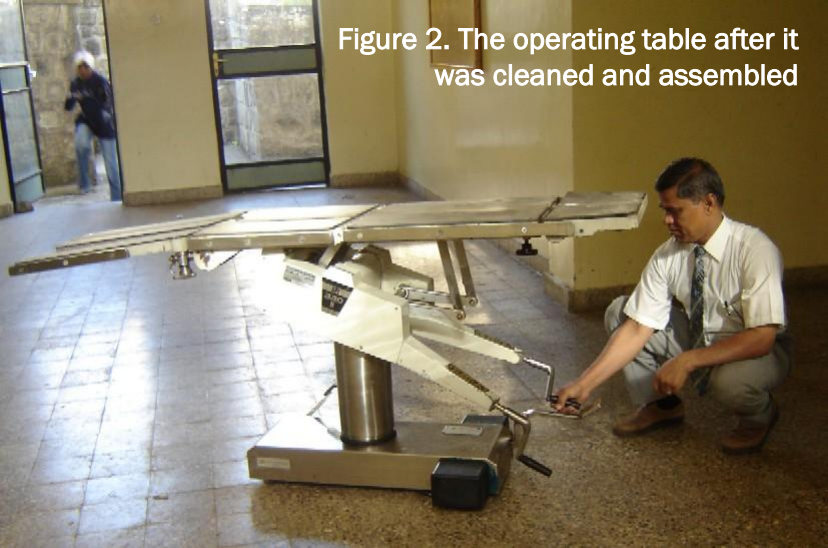
Figure 2. The operating table after it was cleaned and assembled

V Srinivasan

A thoughtful person in the hospital had saved the upholstery, and very soon the table was shifted to the operating theatre for use. Due to the hard work of my colleague Poornachandran and the eight trainees, the hospital now has an extra operating table. Equipment repaired is equipment gained! Some of the lessons learned by the trainees were:

- When a piece of equipment fails, not all parts of it fail. One or at most two parts fail and the instrument stops functioning. Locating the fault is the first step. In the present example, it was the hydraulic system which had failed.
- If a piece of equipment stops functioning, careful examination will determine the problem, which can usually be fixed. Availability of spare parts can be a problem, but this may be solved by using locally available substitutes.

In conclusion, all repairable equipment should be repaired but the will to repair is needed.

**Author's note:** With hydraulic equipment such as this operating table, problems can be prevented by regular maintenance. This simply involves smearing the moving parts, as well as the gasket, with oil on a regular basis. Manufacturers often specify the oil that should be used, but that isn't always available in every country. The author recommends using the oil available for use in the gear boxes of motorcycles or scooters; the same oil can be used to lubricate all other moving parts in the table. The hydraulic system in any instrument should be checked periodically. Any leakage of oil, if noted, should be investigated and stopped.
